# Clinical, Laboratory, Radiological, Bronchoscopic, and Outcome Characteristics of Pulmonary Fungal Infection in Children in PICU in Central China: A Case Series

**DOI:** 10.3389/fped.2022.822043

**Published:** 2022-04-25

**Authors:** Chengjiao Huang, Shuna Xiao, Yin Cheng, Yong Li, Zhi Xia, Wen Tang, Buyun Shi, Chenguang Qin, Hui Xu, Xiaolan Shu

**Affiliations:** Department of Pediatric Intensive Care Unit (PICU), Maternal and Child Health Hospital of Hubei Province, Wuhan, China

**Keywords:** tracheal stenosis, bronchoalveolar lavage fluid, bronchoscopy, invasive fungal infection, child, hospitalized

## Abstract

**Objective:**

Fungal infections are common in pediatric intensive care units (PICUs), but the monitoring methods are limited. This study analyzed the differences in clinical features, diagnosis, and treatment between PICU patients with and without fungal infection.

**Methods:**

This retrospective study analyzed PICU patients at the Maternal and Child Health Hospital of Hubei Province diagnosed with severe pneumonia between January 2015 and January 2020. The patients were divided into the fungal (F) and non-fungal (NF) infection groups. Levels of 1,3-beta-D-glucan (BDG) and galactomannan (GM) in serum and bronchoalveolar lavage fluid (BALF) were analyzed. Chest computed tomography (CT) images were reviewed.

**Results:**

A total of 357 patients were included. In the F group, fever, moist rales, coarse rales, shortness of breath, and sepsis were more common (all *P* < 0.05); PICU time, hospitalization duration, and BDG- and GM-positive rates in serum and BALF were all significantly higher than in the NF group (all *P* < 0.05). The BDG- and GM-positive rates in serum and BALF were higher in the F than in the NF group (all *P* < 0.05). The abnormal lymphocyte ratios in serum were higher in the F group (all *P* < 0.05). Wedge-shaped, patchy, streaky shadows and subpleural reticulation were higher in CT images of the F group (all *P* < 0.05). Tracheobronchial stenosis was more common in pulmonary fibroscopy results of the F group (*P* = 0.04).

**Conclusion:**

PICU pneumonia patients with fungal infection have specific clinical and laboratory features compared with those without fungal infection, including higher rates of BALF, serum BDG, GM positivity and tracheobronchial stenosis.

## Introduction

Fungal infections are common in pediatric intensive care units (PICUs) and neonatal intensive care units (NICUs). Invasive fungal disease is among the main causes of morbidity and mortality of hospitalized pediatric patients, especially premature infants (1–4). Yeast and mold are the most common clinical fungal pathogens. According to a report by the Centers for Disease Control and Prevention, fungal infection is the sixth largest cause of nosocomial infections, and *Candida* spp. is the fourth-most common pathogen responsible for hospital-acquired infections (5). In the NICU, fungal infection is the third most common cause of mortality, with a rate as high as 20–40% (6). In 2000, nosocomial fungal infections in China increased significantly compared with 1993–1996 (24.4 vs. 13.9%) (7). Candidemia ranked fourth in the United States and seventh in Europe among blood infections responsible for the high mortality rate among children (8).

With improvements in diagnostic technologies and treatment methods, the incidence of fungal infections has shown a downward trend in recent years, but its mortality remains high (1, 4). Unfortunately, the methods available to monitor fungal infections are limited and definitive diagnosis in many cases is still difficult. Despite their low sensitivity and long delays before providing results, the fungal culture of blood, body fluids, and respiratory secretions is still considered the gold standard.

Novel methods have shown promise, including serum marker tests such as the *G* test and galactomannan (GM) test, polymerase chain reaction (PCR), matrix-assisted laser desorption/ionization-time of flight, high-throughput pathogen sequencing, and other molecular approaches (9–12). Because the diagnostic accuracy of culture and imaging is low for patients with fungal infections (12, 13), they are often misdiagnosed as tumors, tuberculosis, or inflammatory lesions (14–16), resulting in delayed treatment. Bronchoscopic manifestations and testing of the bronchoalveolar lavage fluid (BALF) in children with pulmonary fungal infections could have a high diagnostic accuracy (17, 18).

Accurate identification and timely diagnosis of fungal infections are crucial to the early control of the disease, as well as reducing medical costs and the economic burden on society and families. Therefore, this paper summarizes the clinical diagnosis and treatment of patients in the PICU of the Maternal and Child Health Hospital from January 1, 2015 to January 1, 2020 and analyzes the general clinical manifestations, chest computed tomography (CT), laboratory examination, fiberoptic bronchoscopy examination, and BALF. The results could provide a reference for medical practitioners.

## Patients and Methods

### Study Design and Patients

This retrospective study examined the data of 357 patients with proven or probable pulmonary fungal infection admitted to the PICU of the Maternal and Child Health Hospital, Hubei Province, between January 1, 2015, to January 1, 2020. All data were prospectively collected in a database. The study was approved by the Ethics Committee of Maternal and Child Health Hospital of Hubei Province (approval number: 2021 IECLW008). Informed consent was waived due to the retrospective nature of the study.

According to the European Organization for Research and Treatment of Cancer/Mycoses Study Group, fungal diagnostic criteria include clinically diagnosed and suspected patients (12, 19). Therefore, the inclusion criteria were (1) patients younger than 18 years and (2) patients who met the diagnostic criteria of severe pneumonia in community-acquired pneumonia (20). Patients with incomplete data, a hospital stay of < 3 days and serum and BALF were not simultaneously tested for 1,3-beta-D-glucan (BDG), and GM were excluded.

### Data Collection and Grouping

**FIGURE 1:**
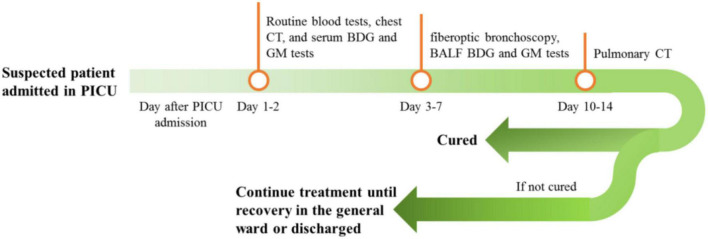
Patient admission process.

Demographic data and clinical characteristics such as clinical manifestation and acute physiology and chronic health evaluation (APACHE) score were obtained from medical records. Routine blood biochemical tests, chest CT, and serum 1,3-beta-D-glucan (BDG) and GM tests were performed on days 1 and 2 of PICU admission. For suspected patients and those with unfavorable outcomes after routine anti-infection treatment, fiberoptic bronchoscopy and alveolar lavage were performed from days 3 to 7; BALF was tested using BDG and GM tests. Chest CT was performed for all patients after 10–14 days of antifungal infection treatment (see Figure 1). All test results and clinical data were recorded and retrospectively analyzed. Patients were divided into fungal (F) and non-fungal (NF) groups, depending on the presence or absence of fungal infection. The diagnostic criteria for fungal infection were (1) the child had a history of cough, wheezing, and fever, (2) pulmonary rales and sounds, (3) no obvious improvement with antibiotic treatment, (4) pulmonary CT showed signs of fungal infection, (5) fungi were cultivated in blood or BALF, (6) the G and GM tests were positive in blood or BALF, and (7) the condition was significantly improved with antifungal treatment (21–23).

### GM and BDG Testing of BALF and Serum

BALF was collected according to the routine bronchoscope operating procedure: 20 mL of normal sterile saline, warmed to body temperature (37°C), was injected for bronchoalveolar lavage. BALF volume of 5–10 mL was recovered and sent for microbial inspection in a disposable sterile silicon plastic bottle. Microbial inspection was performed using an ELx808IU microplate reader (BIoTek, Winooski, VT, United States). The recovered BALF was transferred to a 10-mL centrifuge tube using a disposable sterile pipette. After centrifugation at 1,000 rpm for 10 min using a microfuge 20R centrifuge (Beckman Coulter, Brea, CA, United States), 600–800 μL of supernatant was collected and stored at –40°C for further tests. Venous blood (3 mL) was collected and centrifuged at 3,500 rpm for 10 min; serum was collected and stored at 4°C.

The GM test was performed to detect GM levels in serum and BALF samples using the one-step enzyme immunoassay sandwich method (*Aspergillus* antigen detection kit). The BDG test was mainly performed to detect BDG in serum and BALF samples using the dynamic turbidimetric method (fungal dextran detection kit). All tests were conducted following the manufacturer’s instructions.

Serum GM > 0.5, BALF GM > 0.7, serum BDG > 100 pg/mL, and BALF BDG > 200 pg/mL were defined as positive values for fungal infection (9, 20).

### Chest CT Scan and Interpretation

All patients were placed in the supine position and scanned using a 64-row multi-slice spiral CT. Chest scans were obtained at 1.25-mm intervals using 1.5-mm collimation and were reconstructed with a high-spatial-frequency algorithm. The images were photographed at window settings appropriate for assessing the lung parenchyma (window level, –600 Hounsfield units; window width, 1,600 Hounsfield units). The CT images were retrospectively reviewed by two experienced deputy chief radiologists. The pulmonary nodules or masses, wedge shadow, and consolidation shadow were recorded.

### Fiberoptic Bronchoscopy Examination and Interpretation

Fiberoptic bronchoscopy was performed by a deputy director or a senior doctor using an Olympus fiberoptic bronchoscope. All patients who required bronchoscopy provided informed consent obtained from their parents prior to the treatment (excluding those with interfering factors, such as those receiving antibiotics and antifungal treatment). Routine bronchoscopic alveolar lavage was then performed, and their heart rate, oxygen saturation, and blood pressure were monitored. Under general anesthesia with propofol, 2% lidocaine was locally infused into the larynx *via* the fiberoptic bronchoscope. The top of the fiberoptic bronchoscope was in close contact with the bronchial opening of the lung lobe with abnormal imaging; sterile sodium chloride (0.5–1 mL/kg each time, 15–30 mL in total, at 37°C) was then rapidly injected through the biopsy port. The recovery rate was 40–60%. Furthermore, 50–60 mmHg (1 mmHg = 0.133 kPa) negative pressure suction was applied, and the total amount of lavage fluid was recovered and recorded. The recovered lavage fluid was placed in a uniform sterile container without a heat source and sent for timely inspection.

### Disease Outcome

Pneumonia recovery was defined as the resolution or less cough, wheezing, or fever; lung imaging showed that the infection changes disappeared; the BDG and GM levels became normal. Improved pneumonia was defined as decreased cough and wheezing; lung CT still showed lung shadow (<50%); the GM and BDG levels were partially or completely normal. Uncured was defined as no change or improvement was less than 50%, i.e., lung shadow > 50%.

### Statistical Methods

IBM SPSS statistics 24 (IBM, Armonk, NY, United States) was used for analysis. The categorical variables are presented as frequencies and percentages [*n* (%)] and were compared between groups using the chi-square test or continuous correction method if the effective value was < 5. The continuous variables in accordance with a normal distribution were displayed as mean ± standard deviation (SD) and were analyzed using an independent sample *t*-test; those not in accordance with a normal distribution are displayed as medians (ranges) and were analyzed using the Mann–Whitney *U*-test. *P* < 0.05 was considered statistically significant.

## Results

### General Data and Clinical Features

**FIGURE 2:**
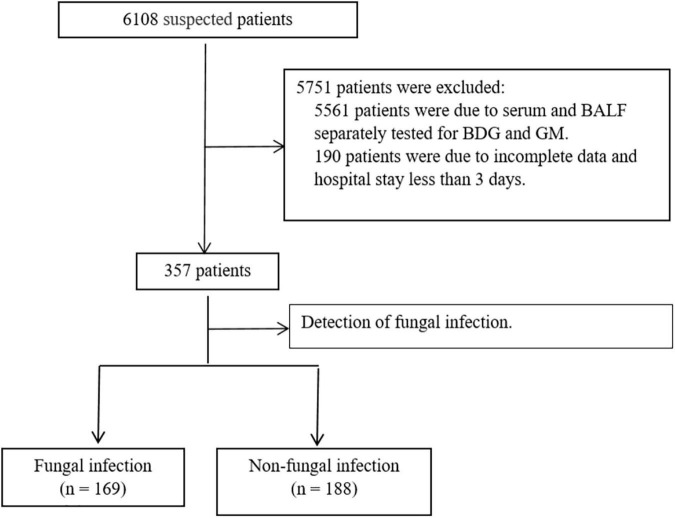
Patient screening process and selection.

Of the total 357 patients in the PICU, 169 were in the F group, and 188 were in the NF group (Figure 2). The incidence of fever (56.2 vs. 42.6%, *P* = 0.01), moist rales (46.2 vs. 33.0%, *P* < 0.01), coarse rales (71.6 vs. 55.9%, *P* < 0.01), shortness of breath (79.3 vs. 63.9%, *P* < 0.01), and sepsis (60.9 vs. 44.7%, *P* < 0.01) were higher in the F group than in the NF group, while wheezing rale (50.9 vs. 67.6%, *P* < 0.01) was lower. Furthermore, the days in the hospital and PICU were significantly increased (days in PICU: 12.80 ± 9.20 vs. 9.91 ± 6.84 days, *P* < 0.01; duration of hospital stay: 19.55 ± 9.29 vs. 15.93 ± 9.23 days, *P* < 0.01; Table 1).

**TABLE 1:** Characteristics of the patients.

Clinical manifestation	F group (*n* = 169)	NF group (*n* = 188)	T	*P*
Male/female	107/62	124/64	0.27	0.60
Age (months)	14.75 ± 21.60	16.46 ± 18.00	1.22	0.22
Weight (kg)	9.24 ± 5.17	10.06 ± 6.48	1.32	0.19
Fever	95 (56.2)	80 (42.6)	6.65	0.01
Fever duration (days)	3.27 ± 7.27	2.45 ± 4.37	1.26	0.21
Cough	153 (90.5)	160 (85.1)	2.43	0.12
Cough duration (days)	9.30 ± 9.83	7.73 ± 8.82	1.58	0.11
Wheezing	54 (32.0)	66 (35.1)	0.40	0.53
Wheezing duration (days)	2.41 ± 5.05	2.70 ± 6.05	0.50	0.62
Distress	56 (33.1)	76 (40.4)	2.03	0.16
Distress duration (days)	0.82 ± 2.43	0.75 ± 1.59	0.36	0.72
Moist rales	78 (46.2)	62 (33.0)	20.17	< 0.01
Coarse rales	121 (71.6)	105 (55.9)	9.50	< 0.01
Wheezing rale	86 (50.9)	127 (67.6)	10.27	< 0.01
Shortness of breath	134 (79.3)	120 (63.9)	10.36	< 0.01
APACHE	98.07 ± 6.46	99.48 ± 6.17	2.11	0.04
Sepsis	103 (60.9)	84 (44.7)	9.44	< 0.01
Septic shock	14 (8.3)	10 (5.3)	1.25	0.26
Mechanical ventilation	44 (26.0)	33 (17.6)	3.79	0.05
Blood purification	6 (3.6)	4 (2.1)	0.24	0.62
Basic diseases				
Premature delivery	22 (13.0)	30 (16.0)	0.62	0.43
Malnutrition	10 (5.9)	5 (2.7)	2.35	0.13
Bronchopulmonary dysplasia	6 (3.6)	3 (1.6)	1.38	0.24
Congenital heart disease	10 (5.9)	9 (4.8)	0.23	0.64
Length of stay (days)	19.6 ± 9.3	15.8 ± 8.2	4.1	< 0.01
Days in PICU (days)	12.8 ± 9.2	9.9 ± 6.8	4.8	< 0.01
Recovery rate	121 (71.6)	125 (66.5)	1.3	0.26
Mortality	2 (1.2)	2 (1.1)	0	> 0.99

*Data are expressed as mean ± standard deviation or n (%).*

*F group, fungal infection group; NF group, non-fungal infection group; APACHE, Acute Physiology and Chronic Health Evaluation scoring system; PICU, pediatric intensive care unit.*

### Results of the BDG and GM Tests

The BDG and GM values of serum and BALF in the F group were significantly higher than those in the NF group (Tables 2, 3). More patients in the F group had positive serum BDG and GM than in the NF group (BDG: 20.7 vs. 5.9%; GM: 11.8 vs. 4.3%; both *P* < 0.01). Similarly, more patients in the F group had positive BALF BDG and GM than in the NF group (BDG: 50.9 vs. 18.6%; GM: 39.1 vs. 17.0%; both *P* < 0.01). Fewer patients in the F group had negative serum BDG and GM than the NF group (67.5 vs. 87.8%, *P* < 0.01). In addition, fewer patients in the F group had negative serum and BALF BDG and GM than the NF group (20.1 vs. 62.8%, *P* < 0.01; Table 4).

**TABLE 2:** Comparison of the serum and BALF BDG levels between groups.

Specimen type	F group (*n* = 169)	NF group (*n* = 188)	T	*P*
Serum BDG (pg/mL)	366.28 ± 386.98	151.63 ± 79.62	4.04	0.03
BALF BDG (pg/mL)	831.91 ± 1185.13	303.67 ± 640.93	5.06	< 0.01

*Data are expressed in mean ± standard deviation.*

*F group, fungal infection group; NF group, non-fungal infection group; BDG, 1,3-beta-D-glucan; BALF, bronchoalveolar lavage fluid.*

**TABLE 3:** Comparison of the serum and BALF GM levels between groups.

Specimen type	F group (*n* = 169)	NF group (*n* = 188)	T	*P*
Serum GM (μg/L)	0.39 ± 1.00	0.18 ± 0.27	2.68	< 0.01
BALF GM (μg/L)	1.30 ± 2.35	0.62 ± 1.17	3.87	< 0.01

*Data are expressed in mean ± standard deviation.*

*F group, fungal infection group; NF group, non-fungal infection group; BALF, bronchoalveolar lavage fluid; GM, galactomannan.*

**TABLE 4:** Comparison of the serum and BALF BDG and GM statuses between groups.

Specimen type	F group (*n* = 169)	NF group (*n* = 188)	T	*P*
Serum BDG +	35 (20.7)	11 (5.9)	17.5	< 0.01
Serum GM +	20 (11.8)	8 (4.3)	7.1	< 0.01
BALF BDG +	86 (50.9)	35 (18.6)	41.4	< 0.01
BALF GM +	66 (39.1)	32 (17.0)	21.7	< 0.01
Serum BDG +, GM +	2 (1.2)	1 (0.5)	0	0.50
Serum BDG-, GM-	114 (67.5)	165 (87.8)	21.5	< 0.01
BALF BDG +, GM +	35 (20.7)	11 (5.9)	17.7	< 0.01
BALF BDG-, GM-	48 (28.4)	130 (69.2)	59.1	< 0.01
Serum BALF BDG +	22 (11.8)	4 (2.1)	14.1	< 0.01
Serum BALF GM +	9 (5.3)	2 (1.1)	4.1	0.04
Serum and BALF BDG +, GM +	1 (0.6)	0 (0)	0	0.96
Serum and BALF BDG-, GM-	34 (20.1)	118 (62.8)	66.2	< 0.01

*Data are expressed in n (%).*

*F group, fungal infection group; NF group, non-fungal infection group; GM, galactomannan; BALF, bronchoalveolar lavage fluid; BDG, 1,3-beta-D-glucan.*

### Pathogens

Results of the mycoplasma, adenovirus, blood culture, sputum culture, and lavage fluid culture showed no significant specificity in the F group compared with the NF group (Table 5).

**TABLE 5:** Comparison of the pathogens found in the two groups.

Pathogens	F group (*n* = 169)	NF group (*n* = 188)	T	*P*
Mycoplasma-positive	68 (40.2)	67 (35.6)	0.80	0.37
Fungi in sputum smear	2 (1.2)	4 (2.1)	0.08	0.78
Fungi in sputum culture	2 (1.2)	1 (0.5)	0.45	0.50
Fungi in BALF culture	1 (0.6)	1 (0.5)	0.01	0.94
Bacteria in blood culture	10 (5.9)	9 (4.8)	0.12	0.73
Bacteria in sputum culture	22 (13.0)	18 (9.6)	1.06	0.30
Bacteria in BALF culture	8 (4.7)	10 (5.3)	0.14	0.71
Adenovirus	14 (8.3)	10 (5.3)	1.25	0.26

*Data are expressed in n (%).*

*F group, fungal infection group; NF group, non-fungal infection group; BALF, bronchoalveolar lavage fluid.*

### Routine Blood Tests

The routine blood test results were compared between the F and NF groups (Table 6). The proportion of C3, serum albumin, prealbumin, prothrombin, and blood monocytes was significantly lower in the F group, whereas the proportion of hydroxybutyric acid, lactate dehydrogenase, and abnormal lymphocytes in blood smear was significantly higher in the F group than that in the NF group (all *P* < 0.05).

**TABLE 6:** Comparison of the routine blood test between groups.

Blood biochemistry	F group (*n* = 169)	NF group (*n* = 188)	T	*P*
Complement C3 (g/L)	0.81 ± 0.22	0.88 ± 0.22	0.89	0.02
Albumin (g/L)	38.33 ± 5.87	40.23 ± 5.20	3.22	0.02
Prealbumin (g/L)	0.13 ± 0.06	0.15 ± 0.07	2.38	0.02
Hydroxybutyric acid (mmol/L)	389.23 ± 236.30	321.91 ± 142.93	3.21	0.01
Lactate dehydro- genase (mmol/L)	508.32 ± 360.63	401.33 ± 206.28	3.39	0.01
Prothrombin time (s)	11.96 ± 1.63	12.34 ± 1.77	2.12	0.03
Mononuclear granulocyte (%)	6.82 ± 3.66	7.87 ± 4.26	2.48	0.02
Abnormal lymphocyte (%)	2.80 ± 4.18	1.93 ± 2.34	2.36	0.02

*Data are expressed as mean ± standard deviation.*

*F group, fungal infection group; NF group, non-fungal infection group.*

### Chest CT Imaging and Fibroscopy of Both Groups

Chest CT imagining revealed that the F group showed a significant increase in wedge-shaped, patchy, streaky shadow and subpleural reticulation (all *P* < 0.05); however, halo sign, cavity, and consolidation did not significantly increase in the F group (Table 7).

**TABLE 7:** Comparison of the chest CT characteristics between groups.

CT changes of lung	F group (*n* = 169)	NF group (*n* = 188)	T	*P*
Patchy, streak	165 (97.63)	160 (85.11)	17.11	< 0.01
Wedge	64 (37.87)	46 (24.47)	7.50	< 0.01
Subpleural	85 (50.30)	66 (35.11)	8.41	0.01
Lumps	17 (10.06)	11 (5.85)	2.18	0.14
Interstitial	43 (25.44)	33 (17.55)	3.31	0.07
Halo sign	1 (0.59)	0 (0)	0.01	0.97
Cavitation	7 (4.14)	1 (0.53)	3.76	0.05
Consolidation	39 (23.08)	29 (15.42)	3.38	0.07
Bronchial infiltration	20 (11.83)	14 (7.45)	1.99	0.16

*Data are expressed in n (%).*

*F group, fungal infection group; NF group, non-fungal infection group; CT, computed tomography.*

Pulmonary fibroscopy findings were compared between both groups (Table 8). Changes in the bronchoscopy results observed in the F group could contain bronchial congestion, edema, paleness, necrosis, and bleeding. The lavage fluid could contain pus and blood and have a sticky, jelly, or foamy consistency. Tracheobronchial stenosis was more common in the F group than in the NF group (55.0 vs. 44.1%, *P* = 0.04). There was no significant difference in the properties of lavage fluid and tracheomalacia between the two groups.

**TABLE 8:** Comparison of the pulmonary fibroscopic findings between groups.

Changes under fiberscope	F group (*n* = 169)	NF group (*n* = 188)	T	*P*
Hyperemia and edema	158 (93.49)	172 (91.49)	0.51	0.48
Pale	5 (2.96)	6 (3.19)	0.02	0.90
Purulent fluid	141 (83.43)	155 (82.45)	0.06	0.81
Mucus	21 (12.42)	19 (10.11)	0.48	0.49
Peptone like	5 (2.96)	8 (4.26)	0.43	0.51
Mucosal necrosis	4 (2.37)	5 (2.66)	0.01	> 0.99
Plastic bronchitis	0 (0)	1 (0.53)	0.01	> 0.99
Tracheobronchial stenosis	93 (55.03)	83 (44.15)	4.22	0.04
Tracheobronchial malacia	38 (22.49)	39 (20.74)	0.16	0.69
Anomalous branching	6 (3.55)	5 (2.66)	0.24	0.63

*Data are expressed in n (%).*

*F group, fungal infection group; NF group, non-fungal infection group.*

## Discussion

Invasive fungal infection is an important cause of morbidity and mortality in children, especially infants and premature neonates in a hospital setting. However, prompt and definitive diagnosis is challenging because of non-specific symptoms and inadequate detection tests. Although the present study was not a formal diagnostic study that could suggest replacing the gold standard for fungal infection, the results might suggest that using antifungal therapies combined with tests like serum and BALF BDG and GM could enable a timely diagnosis of pulmonary fungal infections. An early diagnosis should improve the prognosis of pediatric patients, but prospective studies are necessary for confirmation. Here, we analyze these options with the aim of providing a reference for clinicians as well as future studies.

There was no significant difference between the F and NF groups with respect to preterm birth, malnutrition, congenital heart disease, and pulmonary dysplasia, among others. The duration of hospital and PICU stay of patients with fungal infections was significantly longer, which is consistent with previous studies (8); however, this may have been due to the time required for GM detection.

At our hospital, the GM detection results were obtained in 3–4 days and BDG in 2 days. It is suggested that fungal detection and assessments should be conducted at the earliest time possible to reduce the duration of hospital stay and thereby reduce the spread of nosocomial infections. Fever was a common symptom in patients with fungal infections. Moist rales, coarse rales, wheezing, and dyspnea were common clinical symptoms in patients with fungal infections than in those with non-fungal infections; this appears to be similar to the observations by Wu et al. (24). Therefore, if the fever is unexplained and conventional therapy proves to be challenging to treat pulmonary rales and dyspnea, a diagnosis of fungal infection should be promptly considered.

Our results revealed a significantly higher incidence of sepsis in the F group (Table 1), suggesting that fungal sepsis should be considered in addition to bacterial sepsis during diagnosis. Furthermore, the levels of non-specific indicators such as C3, albumin, prealbumin, hydroxybutyrate, lactate dehydrogenase, prothrombin, monocyte ratio, and abnormal lymphocyte ratio were higher in the F group than in the NF group (Table 5); and the APACHE score was significantly lower in the F group. These data are consistent with those by Li et al. (25). Therefore, it is suggested that fungal infection should be considered in addition to bacterial infection during clinical diagnosis since it can have serious inflammatory reactions and clinical manifestations. Prompt identification of the pathogen is vital in cases of severe sepsis.

Singhi et al. (26) conducted a prospective study with 186 PICU patients and found that 45 (69%) had *Candida* colonization. Oropharyngeal (52%) and rectal (43%) colonization were more common than that of the skin (34%). The species identified were *Candida tropicalis* (34.2%), *C. parafilariasis* (28.8%), and *C. albicans* (14.4%). Twenty patients (30.2%) had candidemia; among these, 18 (90%) showed the presence of colonization, and 15 (75%) had active *Candida* infection. It is suggested that patients with sepsis and septic shock who have been treated in the PICU for > 5 days should be promptly tested for *Candida* colonization, especially children with central venous catheters. Sepsis, septic shock, and fungal infection may interact and induce either of the illnesses.

In this study, the mortality rates in the F and NF groups were 1.18% (2/169) and 1.06% (2/188), respectively. This is not consistent with the mortality rates of 14.4% reported by Warris et al. (27) and 29.4% reported by Chakrabarti et al. (28) in India. However, there were no patients with tumors or transplants at our hospital; therefore, we are unable to entirely represent the global data for mortality rates. Moreover, these low rates may be due to increased awareness and technological improvements in fungal diagnostic methods in recent years and early antifungal treatment. Another reason for this could be that we excluded patients with a hospital stay of < 3 days; thus, we automatically excluded the number of deaths. Therefore, prompt diagnosis and treatment of fungal infection can significantly reduce mortality and improve the treatment rate.

Similar to other studies, our study showed that the absolute values and specific positive rates of BDG and GM in the serum and BALF of patients with fungal infections were significantly higher than those of patients without fungal infections, suggesting that these parameters remain suitable for the clinical diagnosis of fungal infections (29). Tong et al. believe that continuous observation of G and GM testing can guide clinical practice to some extent (29). In our study, positive rates of BDG and GM in BALF (50.38 and 39.05%, respectively) were significantly higher than those in serum (21.71 and 11.83%, respectively), and the simultaneous positive rates of BDG in serum and BALF were 11.83% (22/169) and 5.33% (9/188), respectively, which were significantly higher in the F group than in the NF group. It supports the argument that BDG and GM are both suitable in the clinical diagnosis of fungal infections, especially when combined with existing detection methods such as chest CT, which has a high diagnostic value (30).

Although new detection methods such as PCR, high-throughput pathogen sequencing, and mass spectrometry (31, 32) have been used for the diagnosis of fungal infection in recent years, they have limitations in clinical use because of cost among other factors. However, some studies have found that the combination of chest CT and new detection methods can greatly improve the detection rate (13, 15). Although there is no specificity in CT manifestations of fungal infections, it can guide clinicians to determine the presence of a fungal infection. However, previously reported manifestations such as crescent signs, halo signs, and cavity and other signs (12, 13) specific to fungal infections were not common in our group of patients; these findings of our study are consistent with those by Burgos et al. (31). In contrast, non-specific manifestations such as wedge shadow and subpleural reticulation were common in the F group. However, in this group, the author observed that a combination of the patients’ history, physical characteristics, chest CT changes, and serum and BALF BDG and GM tests could help detect fungal infections. Moreover, specific factors such as hospitalization before admission, history of antibiotic treatment and hormone, and treatment outcome of pulmonary rales with antibiotics often suggest the presence of fungal infection. CT diagnosis of children with pulmonary fungal infections is complex, and the lesions are widely distributed, showing pleomorphism, multifocality, and variability (7). For the same disease with different shadows, the same shadow of different diseases should be carefully identified. Whenever necessary, more advanced detection methods such as positron emission tomography/magnetic resonance imaging (15) should be performed to increase the accuracy of diagnosis, for timely diagnosis and treatment, and to reduce complications and mortality.

In Table 7, fibroscopy results showed that secretions were not specific in the F group, and fungal infection could not be clinically detected only from sputum. However, the incidence of tracheobronchial stenosis in the F group was significantly higher than that in the NF group (55.0 vs. 44.1%), suggesting that tracheobronchial infection can be accompanied by stenosis or be associated with fungal infection. Marchioni et al. (33) analyzed the changes in the bronchi lumen of 2,075 adults for 10 years. Among these, 22% showed tracheobronchial stenosis, with *Aspergillus* being the secondary pathogen of endobronchial diseases requiring medical treatment. Fifty-three cases of tracheobronchial fungal infection (tracheobronchial mucormycosis) in adults showed tumor-like changes, and the most common characteristics were broncho-obstruction due to tumor, mucosal necrosis, uneven mucosa, or a mass and bronchial stenosis with mucosal hyperplasia or mucosal heterogeneity (34). The causes of tracheobronchial stenosis in children with mycotic infection of the trachea and bronchus may be related to the inflammatory injury caused by the infection (35) or invasive procedures such as tracheal intubation (36) and bronchoscopy (37), among others. Though children did not report infection with tracheobronchial fungi after bronchofibroscopy in this study, care still should be taken while performing clinical procedures to avoid tracheal stenosis and re-invasion.

Our study is promising, but it has some limitations. Most fungal diagnoses are clinical diagnoses, and diagnostic procedures, including fungal culture-based methods, have low yield. Furthermore, ours is a retrospective study; therefore, although our findings are significant, the data obtained with our limited sample size may be insufficient. Not all patients were tested for BDG and GM after treatments, and the data were too incomplete for analysis. A larger sample size analyzing prospective studies may provide robust data. Moreover, there is no test clearly indicating the presence of colonization or active fungal infection, which may lead to other complications caused by excessive antifungal treatment.

## Conclusion

The factors associated with fungal infections should be considered when evaluating pediatric patients. PICU pneumonia patients with fungal infection have specific clinical and laboratory features compared with those without fungal infection, including higher rates of BALF, serum BDG, GM positivity and tracheobronchial stenosis. Using antifungal therapies combined with tests like serum and BALF BDG and GM could enable a timely diagnosis of pulmonary fungal infections, possibly improving prognosis. Future prospective studies should examine the diagnosis and prognosis of PICU pneumonia patients with fungal infection.

## Data Availability Statement

The original contributions presented in the study are included in the article/supplementary material, further inquiries can be directed to the corresponding author/s.

## Ethics Statement

The studies involving human participants were reviewed and approved by the Ethics Committee of Maternal and Child Health Hospital of Hubei Province (approval number: 2021 IECLW008). Written informed consent to participate in this study was provided by the participants’ legal guardian/next of kin.

## Author Contributions

CH and SX collected the data on all fungal infections. HX analyzed and explained the data of patients with fungi. CH was a major contributor to the manuscript. All authors read and approved the final manuscript.

## Conflict of Interest

The authors declare that the research was conducted in the absence of any commercial or financial relationships that could be construed as a potential conflict of interest.

## Publisher’s Note

All claims expressed in this article are solely those of the authors and do not necessarily represent those of their affiliated organizations, or those of the publisher, the editors and the reviewers. Any product that may be evaluated in this article, or claim that may be made by its manufacturer, is not guaranteed or endorsed by the publisher.

## Abbreviations

PICUs: pediatric intensive care units
NICUs: neonatal intensive care units
F group: fungal infection group
NF group: non-fungal infection group
BDG: 1,3-beta-D-glucan
GM: galactomannan
BALF: bronchoalveolar lavage fluid
CT: computed tomography
PCR: polymerase chain reaction
APACHE: acute physiology and chronic health evaluation.
